# good Outcomes of Combined Femoral Derotation Osteotomy and Medial Retinaculum Plasty in Patients with Recurrent Patellar Dislocation

**DOI:** 10.1111/os.12500

**Published:** 2019-08-16

**Authors:** Guang‐min Yang, Yan‐yang Wang, Li‐xiong Zuo, Fa‐quan Li, Yi‐ke Dai, Fei Wang

**Affiliations:** ^1^ Department of Joint Surgery Third Hospital of Hebei Medical University Shijiazhuang Hebei China; ^2^ Department of Radiology Third Hospital of Hebei Medical University Shijiazhuang Hebei China

**Keywords:** Combined operation, Femoral anteversion, Femoral derotation osteotomy, Recurrent patellar dislocation, Rotational malalignment

## Abstract

**Objectives:**

To report the clinical outcomes of combined femoral derotation osteotomy and medial retinaculum plasty for recurrent patellar dislocation in patients with excessive femoral anteversion.

**Methods:**

From January 2015 to March 2018, 20 knees in 20 patients (18 female, 2 male) with a mean age of 21 ± 4.2 years (range, 16 to 28 years) were retrospectively reviewed. All patients had undergone femoral derotation osteotomy and medial retinaculum plasty for recurrent patellar dislocation and excessive femoral anteversion angle (FAA > 25°). CT and X‐rays were used to assess the correction of the femoral anteversion angle, the tibia tuberosity‐trochlear groove (TT‐TG) distance, patellar tilt, and the congruence angle following the combinatory operations. Subjective scores, such as Kujala, International Knee Documentation Committee (IKDC), Tegner, and visual analogue scale (VAS) scores, were used to evaluate knee function preoperatively and postoperatively.

**Results:**

No recurrence of patellar dislocation occurred in these patients during an average of 18 months (range, 12 to 23 months) of follow‐up. The mean of the FAA was corrected to 15.80° ± 3.58° postoperatively compared with 31.42° ± 4.95° preoperatively (*P* < 0.001). The TT‐TG distance was decreased from 22.17 ± 5.28 mm before surgery to 19.42 ± 4.57 mm after surgery (*P* = 0.03). The patellar tilt and congruence angle were improved from 30.43° ± 5.30°, 43.30° ± 11.04° to 15.80° ± 3.94°, 16.64° ± 9.98°, respectively (*P* < 0.001). The Kujala score was improved from 72.4 ± 19.90 before the surgery to 88.2 ± 12.25 after the surgery (*P* < 0.001). The IKDC score was improved from 70.56 ± 21.44 to 90.78 ± 14.32, and the VAS score was decreased from 4.23 ± 2.11 preoperatively to 1.27 ± 1.08 postoperatively (*P* < 0.001). No significant difference in Tegner score (5.46 ± 2.49 *vs* 5.79 ± 1.44) was found before and after the surgery (*P* = 0.2). Patients younger than 20 years old had lower Kujala (83.46 ± 14.56 *vs*. 90.84 ± 7.74, *P* = 0.02) and IKDC (83.49 ± 17.35 *vs* 92.46 ± 9.28, *P* = 0.04) scores than those older than 20 years.

**Conclusion:**

Good knee function, pain relief, and improved patellofemoral congruence were achieved with the combined femoral derotation osteotomy and medial retinaculum plasty. The combined operations serve as an ideal treatment for recurrent patellar dislocation and address the primary risk factors.

## Introduction

Patellar instability is an important factor affecting athletic ability that can occur at a young age. According to research by Fithian *et al*.^1^ and Atkin *et al*.^2^, 69% of patellar dislocations occur between the ages of 10 and 19 years. Moreover, the rate grew even higher in the next decades of their life. Stefancin *et al*.^3^ found a near 48% recurrence rate in individuals who had undergone non‐operative treatments. The etiology, diagnosis, and surgical treatments of recurrent patellar dislocation (RPD) have been progressing in the past fifteen years. However, there is still uncertainty regarding the anatomical risk factors which influence the treatments for RPD.

Recurrent patellar dislocation is generally considered to be caused by malalignment between the trochlear groove and the patellar tracking. In the past fifteen years, the operative strategies for correcting the congruence of patellofemoral joint have demonstrated better outcomes both in terms of patients’ satisfaction and the radiological assessments following single or combined surgery. The congruence of the patellofemoral joint was dependent on the relative position of the femoral trochlea and patellar tracking, and was affected by several predispositions both in the osseous and soft tissue deformities described in the previous studies^4^. The femoral derotation osteotomy involved surgical external rotation of the distal femur to adjust the patellofemoral congruence. The internal rotation of the distal femur was assumed in previous studies to be the major cause of the rotational malalignments in the patellofemoral joint. In the research by Diederichs *et al*.^5^ and Franciozi *et al*.^6^, a greater femoral anteversion was found in the patients with a history of patellar dislocation, and greater femoral internal rotation was demonstrated to have negative effects on the clinical outcomes of the patellar stability surgery.

Besides the osseous abnormalities, the tracking of the patella was also influenced by the medial retinaculum, which provides the static and dynamic stability in the patellofemoral joint^7^. The medial patellar soft tissue was tended to slack or even ruptured under the sustained lateralization and lateral force vector of the patella. Both biomechanical studies and clinical research have proved the effectiveness and good outcomes of medial patellar soft tissue repair or reinforcement against the patellar dislocation^7^, ^8^, ^9^. Operations repairing the medial structure now accept it as a principle method for RPD patients with or without malalignments. Although the surgical management of patellar instabilities improved in recent years, internal femoral rotation remains as the primary risk factor of patellar dislocation and is rarely corrected by the surgery^6^, ^10^, ^11^.

The rotational malalignment of the lower limb is considered to be the primary risk factor of recurrent patellar dislocation for patients with higher femoral anteversion^12^, ^13^. In previous research, combined operations for correcting osseous abnormalities and soft tissue have demonstrated better outcomes than the single operation^10^, ^14^. In this study, we performed a femoral derotation osteotomy in patients with greater femoral anteversion to correct the rotational deformities on the distal femur and restore the medial stability of the patella through medial retinaculum plasty. We hypothesized that the combined operation could correct the osseous and soft tissue abnormalities and improve the function of the knee joint in patients with recurrent patellar dislocation and excessive femoral anteversion.

## Materials and Methods

### *Patients*

From January 2015 to March 2018, a total of 20 patients (20 knees) with recurrent patellar dislocation were retrospectively reviewed in this study. All patients received combined surgery of femoral derotation osteotomy and medial retinaculum plasty to correct osseous and soft tissue abnormalities. This study and detailed surgical procedures were approved by the ethical committee of the hospital.

The inclusion criteria were: (i) recurrent patellar dislocation (≥3 times); (ii) femoral anteversion angle (FAA) > 25°; (iii) failure of non‐operative treatments; and (iv) Dejour type A or B trochlear dysplasia. The exclusion criteria were: (i) body mass index (BMI) > 30 kg/m^2^; (ii) patellar chondromalacia or patellofemoral arthritis; (iii) less than 16 years old and with open epiphyseal plates; (iv) previous ligament rupture or ligament stabilization surgery; and (v) Dejour type C or D trochlear dysplasia.

### *Surgical Techniques*

#### *Anesthesia and Position*

All surgical procedures were performed by the same surgical team. The patients received spinal anesthesia and were placed in the supine position. A tourniquet was tied to the proximal thigh and set to 280 mmHg. The aseptic surgical area was prepared using iodine (2%) and medical alcohol (70%).

#### *Approach*

**Figure 1:**
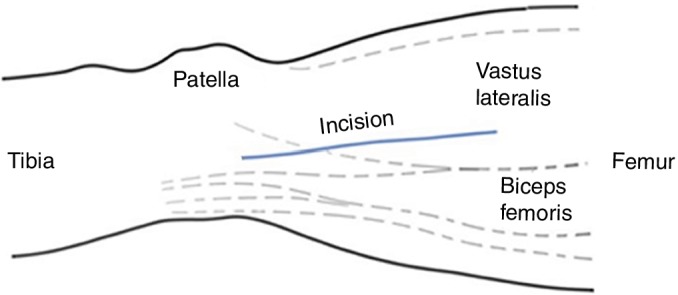
Schematic of the surgical incision. The lateral approach parallel to the axis of the distal femur was chosen to expose the distal femoral shaft through the intermuscular space. The dotted line indicated the border of the vastus lateralis muscle and the biceps femoris muscle.

A 10‐cm incision was made along the longitude axis of the distal femur on the lateral thigh (Fig. 1). The subcutaneous tissue and fascia were carefully separated. The intramuscular space between the lateral border of vastus lateralis and biceps femoris was incised to expose the distal femoral shaft.

#### *Osteotomy and Reconstruction*

**Figure 2:**
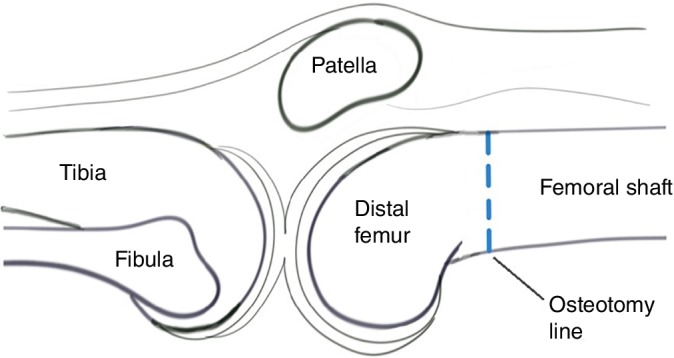
Schematic of the femoral supracondylar osteotomy. The osteotomy line was marked parallel to the tibiofemoral joint line above the femoral condyle. After osteotomy, the distal femur was externally rotated to eliminate excessive femoral anteversion.

**Figure 3:**
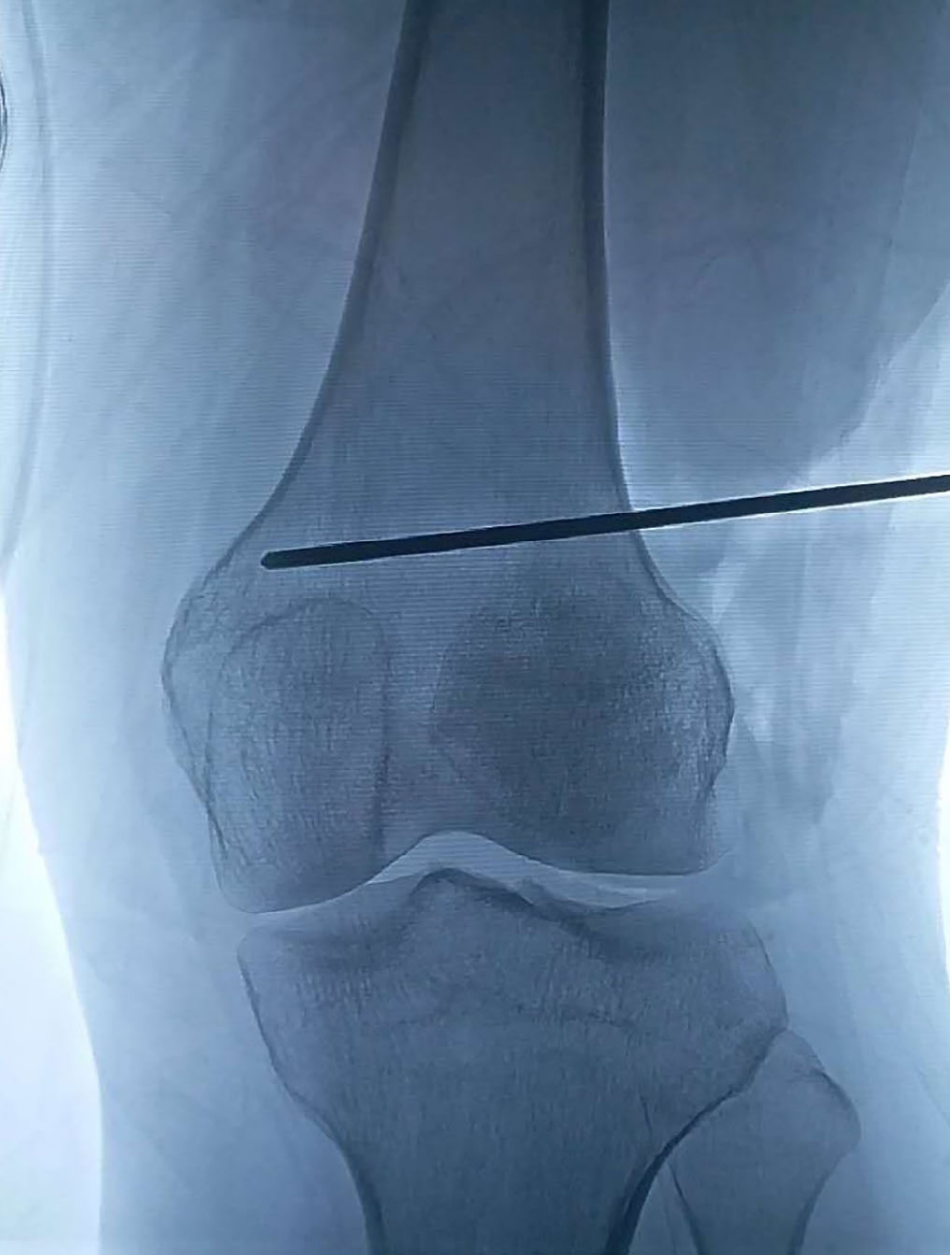
Intraoperative fluoroscopy of the distal femur. Before the osteotomy, a guide pin parallel to the joint line was placed above the femoral condyle to orient the osteotomy line.

**Figure 4:**
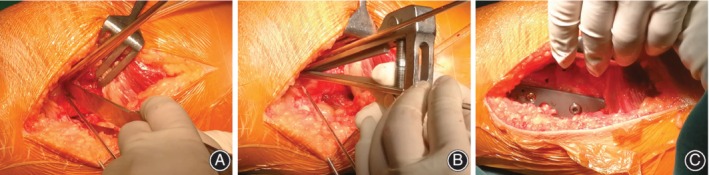
Detailed procedure of derotation osteotomy. (A) The osteotomy was performed according to the direction of the guide pin. (B) Derotation angle between the 2 K‐wires was adjusted according to the preoperative assessments. (C) The distal femur was fixed using a lateral femoral plate.

A schematic of the femoral supracondylar osteotomy is shown in Fig. 2. Two Kirschner wires were inserted as a marker of the supracondylar osteotomy line, which was parallel to the knee joint line under the intraoperative fluoroscopy (Fig. 3). Two Kirschner wires were inserted into both sides of the osteotomy line to mark the rotation degree. The femoral shaft was transected by the swing dust and bone chisel (Fig. 4A). The distal femur was externally rotated to adjust the rotational alignments of the patellofemoral joint according to the preoperative assessments. Two terminals of the transected femoral shaft were temporarily fixed by a Kirschner wire oblique crossing the femoral shaft (Fig. 4B). Fluoroscopy was used to ensure the reduction of the femoral shaft. Then one lateral femur plate was placed to definitively fix the femoral shaft (Fig. 4C).

#### *Medial Retinaculum Plasty*

**Figure 5:**
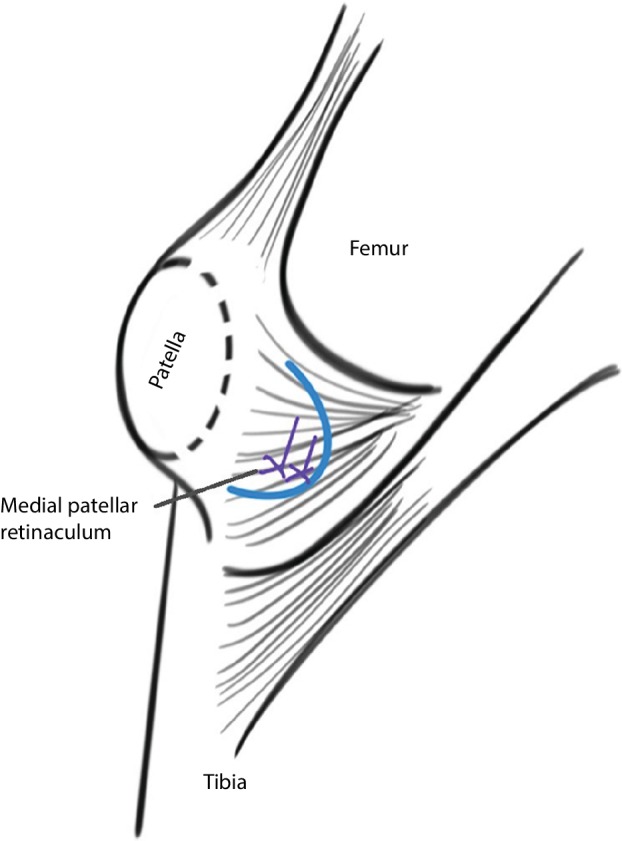
Schematic of the medial retinaculum plasty. To repair the medial soft tissue of the patella, the medial patellar retinaculum was dissected and strengthened using high strength wire or anchor on the femoral side.

**Figure 6:**
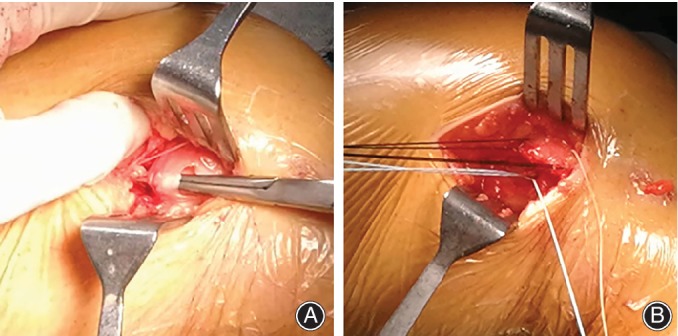
Detailed procedure of medial retinaculum plasty (A) The medial patellar retinaculum on the femoral side was separated. (B) The insertion of the medial patellar retinaculum (medial structure) was repaired and sutured on the femoral side using high strength wire or anchor.

A schematic of the operation is presented in Fig. 5. For the medial retinaculum plasty, a 3‐cm incision was made on the medial thigh between the patella and the medial condyle. After separating the subcutaneous tissue, the medial retinaculum close to the medial condyle was dissected (Fig. 6A). The position of the patella was changed by adjusting the tension of the medial retinaculum. Then the insertion of medial retinaculum was stitched through the cortex of medial condyle using high strength wire (Rigidfix, Ethibond, Johnson & Johnson, USA) (Fig. 6B). In 14 of 20 patients, the femoral insertion was fixed by one anchor (Twinfix, Smith & Nephew, USA) at the anatomical point of medial patellofemoral ligaments according to previous studies^15^, ^16^. After these procedures, the subcutaneous tissue and skin were stitched up.

### *Rehabilitation*

A restrictive brace for the lower limbs was used right after the surgery. In the hospital, mild weight‐bearing exercises were allowed with the help of crutches. The patients started straight leg raising in the hospital. Partial weight‐bearing exercises were recommended and gradually increased at 6 weeks after the surgery. Passive and independent knee flexion was carried out during this period. After 12 weeks, patients were advised to stop using crutches and return to daily activity; gentle exercises were recommended depending on the healing of the osteotomy line.

### *Radiological Assessments*

Detailed radiological assessment of lower limbs, including weight‐bearing X‐rays and CT scans, were conducted preoperatively and postoperatively.

#### *Femoral Anteversion Angle*

**Figure 7:**
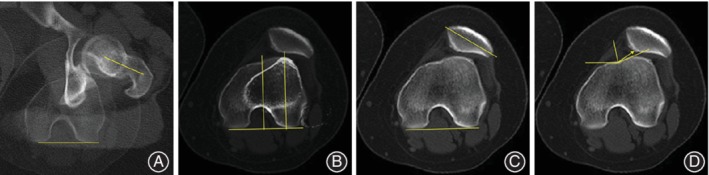
The preoperative measurements under axial CT images. (A) Superimposed images in the sections of the femoral neck and bilateral femoral condyle showing the femoral anteversion angle. (B) Superimposed images in the sections of the bilateral femoral condyle and tibial tuberosity showing the tibia tuberosity‐trochlear groove distance. (C) patellar tilt. (D) congruence angle.

In the study of Dejour *et al* 4, the FAA in the patients with patellar dislocation was significantly higher (>15°) than in controls. In this study, the change of the FAA before and after the surgery was used to evaluate the effectiveness of the femoral derotation osteotomy. The femoral anteversion angle was measured in the axial CT images according to the study of Franciozi *et al*.^6^ A line through the center of the femoral head and the femoral neck axis was drawn. Another line was drawn crossing the lowest point of the medial and lateral femoral posterior condyles. The angle of the two lines was defined as the femoral anteversion angle (Fig. 7A).

#### *Tibia Tuberosity‐Trochlear Groove Distance*

The tibia tuberosity‐trochlear groove (TT‐TG) distance was frequently used to evaluate the lateral force vector of the patella, and an increased TT‐TG distance (≥20 mm) was the leading risk factor for patellar dislocation according to the study of Schoettle *et al*.^17^ The TT‐TG distance was measured using axial CT images. A line perpendicular to the posterior condyle line and through the lowest point of the trochlear groove was drawn. Another line through the tibial tubercle and perpendicular to the posterior condyle line was drawn. The distance between the two lines was defined as TT‐TG distance (Fig. 7B).

#### *Patellar Tilt*

Compared with the controls, a patellar tilt more than 20° indicated abnormal patellar tracking in patients with patellar dislocation^4^. The value of the patellar tilt was measured using axial CT images. It was defined as the angle between the line of the maximal axis of the patella and the posterior condyle line (Fig. 7C).

#### *Congruence Angle*

According to the study of Merchant *et al*., an excessive congruence angle (>16°) existed in 95% of patients with patellar subluxation, which indicated the lateralization of the patellar from the trochlear groove^18^, ^19^. The congruence angle was measured using the radiographs (sunrise view) according to Merchant *et al* 18. A line was drawn through the lower pole of the patellar and the deepest point of the trochlear sulcus. The angle of the line lateral to the bisector of the trochlear angle was defined (Fig. 7D). This angle is negative on the medial of the bisector line and positive on the lateral side.

### *Clinical Assessment*

The visual analogue scale (VAS) score was used to evaluate the pain relief postoperatively. Subjective function scores, including the Tegner sports score, the Kujala score, and the International Knee Documentation Committee (IKDC) score, were used to evaluate the improvement in knee function^20^, ^21^. The clinical assessments postoperatively were collected at last follow‐up and compared with the preoperative assessments.

### *Statistical Analysis*

The radiological variables and clinical scores are shown as median and SD values. The significance of the radiological variables and the clinical scores were analyzed by paired Student *t*‐test. The data were measured by two different surgeons. A *P*‐value less than 0.05 was considered statistically significant. All analyses were performed using SPSS software (Version 22.0, IL, USA).

## Results

### *Demographics*

From 2015 to 2018, a total of 20 patients (18 women and 2 men) underwent unilateral surgery. The average follow‐up time was 18 months (range from 12 to 23). The average age of the patients at the time of surgery was 21 ± 4.2 years old (range, 16 to 28 years), and the average BMI was 25.24 ± 4.20 kg/m^2^.

### *Surgery and Complications*

The mean length of the surgery was 50 min (range, 40–65 min) and blood loss was 70 mL (range, 60–90 mL). No patients had postoperative wound infection, hemorrhage, or related nerve injury. There was no recurrence of patellar dislocation at the last follow‐up. Two patients had occasional clicking of the knee joint during the knee flexion but no pain and limitation in the follow‐up. One patient had a limited knee flexion 2 months after the surgery; the limitation improved in the following 1 month after enhanced passive exercises. Three patients felt pain in the lateral thigh, and the pain was gradually relieved with daily activity. All patients were satisfied with the outcomes and returned to physical activities 8 months after the surgery.

### *Radiological Outcomes*

**Figure 8:**
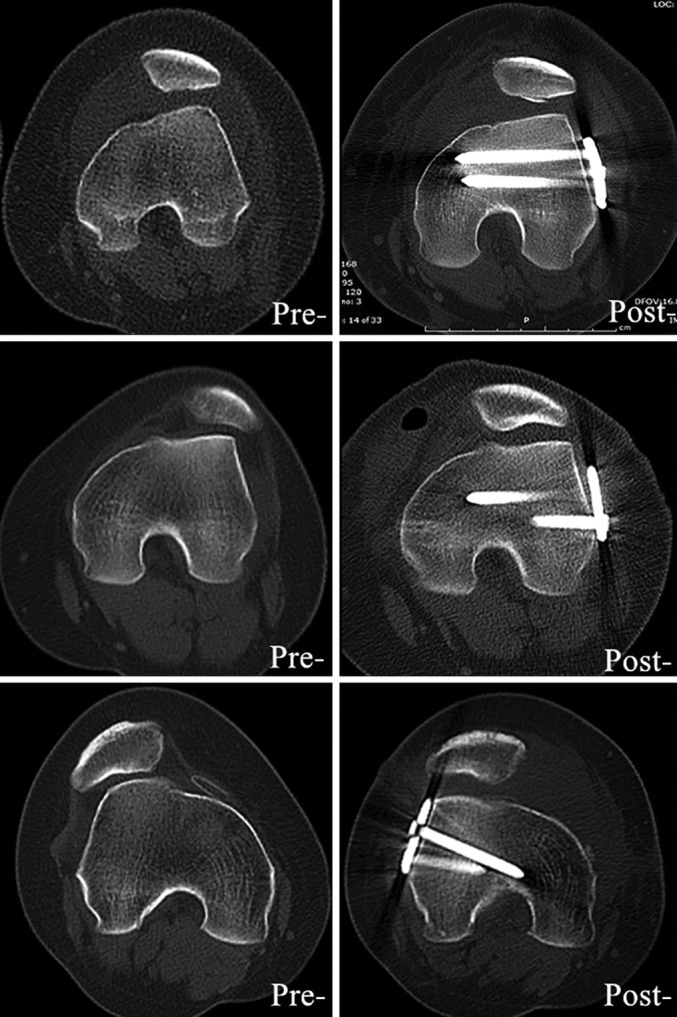
The postoperative axial CT images of three young female patients were presented to show the improved patellofemoral congruence. The patella position after the surgery was centralized compared with the preoperative.

Preoperative and postoperative radiological images are presented to show the improved patellofemoral congruence of the RPD patients (Fig. 8). The postoperative radiological outcomes were obtained approximately 6 months after the surgery and compared with the preoperative (Table 1).

**Table 1:** Preoperative and postoperative radiographic outcomes of the rotational alignment of the patellofemoral joint

Indexes	Preoperation		Postoperation		*P‐*value
	Mean	SD	Mean	SD	
FAA (°)	31.42	4.95	15.80	3.58	*P* < 0.001
TT‐TG (mm)	22.17	5.28	19.42	4.57	*P* = 0.03
PT (°)	30.43	5.30	15.80	3.94	*P* < 0.001
CA (°)	43.30	11.04	16.64	9.98	*P* < 0.001

CA, congruence angle; FAA, femoral anteversion angle; PT, patellar tilt; SD, values were reported as mean with standard deviation; TT‐TG, tibia tuberosity‐trochlear groove distance.

#### *Femoral Anteversion Angle*

Compared with preoperatively, the femoral anteversion measured by the FAA was significantly improved postoperatively. The average FAA before the surgery in 20 patients was 31.42° ± 4.95°, which was significantly higher than the normal value (15°). After the surgery, the FAA was reduced to 15.80° ± 3.58°, which was close to the normal level (*P* < 0.001).

#### *Tibia Tuberosity‐Trochlear Groove Distance*

The average TT‐TG distance before the surgery in 20 patients was 22.17 ± 5.28 mm, which was significantly higher than the threshold value (20 mm). The TT‐TG distance was reduced to 19.42 ± 4.57 mm preoperatively, with an average correction of 2.75 mm (*P* = 0.03).

#### *Patellar Tilt*

Before the surgery, the average patellar tilt in 20 patients was 30.43° ± 5.30°, which was significantly higher than for a healthy knee (20°). After the surgery, the average patellar tilt was corrected to 15.80° ± 3.94° (*P* < 0.001).

#### *Congruence Angle*

The average congruence angle was decreased from 43.30° ± 11.04° preoperatively to 16.64° ± 9.98° postoperatively, with an average improvement of 26.7° (*P* < 0.001).

### *Clinical Outcomes*

In terms of clinical outcomes, the pain and function of the knee joint were significantly improved postoperatively (Table 2). The VAS score was decreased from 4.23 ± 2.11 preoperatively to 1.27 ± 1.08 postoperatively (*P* < 0.001). The mean Kujala score was significantly improved from 72.4 ± 19.90 preoperatively to 88.2 ± 12.25 postoperatively (*P* < 0.001). The average IKDC score was significantly improved from 70.65 ± 21.44 preoperatively to 90.78 ± 14.23 postoperatively (*P* < 0.001). No significant difference in the Tegner score (5.46 ± 2.49 *vs* 5.79 ± 1.44) was found before and after the surgery (*P* = 0.2).

**Table 2:** Comparison of preoperative and postoperative functional scores

Scores	Preoperative		Postoperative		*P*‐value
	Mean	SD	Mean	SD	
VAS	4.23	2.11	1.27	1.08	*P* < 0.001
Tegner	5.46	2.49	5.79	1.44	*P* = 0.2
Kujala	72.4	19.90	88.2	12.25	*P* < 0.001
IKDC	70.56	21.44	90.78	14.32	*P* < 0.001

IKDC, International Knee Documentation Committee; SD, functional scores were reported as mean with standard deviation; VAS, visual analogue scale.

### *Variables for Poor Functional Scores*

Preoperative age, classification of trochlear dysplasia, and TT‐TG distance were analyzed to explore the potential factors affecting the postoperative outcomes. The results are presented in Table 3.

**Table 3:** Outcomes according to the preoperative variables

Variables	Tegner	*P*‐value	Kujala	*P*‐value	IKDC	*P*‐value
Year ≤20	5.34 ± 1.66	0.23	83.46 ± 14.56	0.02	83.49 ± 17.35	0.04
Year >20	6.21 ± 2.01		90.84 ± 7.74		92.46 ± 9.28	
Type A	5.96 ± 1.60	0.53	86.15 ± 11.30	0.16	92.37 ± 14.30	0.21
Type B	5.14 ± 1.79		92.47 ± 10.41		88.18 ± 10.44	
TT‐TG <20 mm	5.45 ± 1.85	0.32	89.38 ± 8.57	0.67	91.05 ± 14.20	0.19
TT‐TG >20 mm	6.28 ± 2.34		84.27 ± 16.21		88.37 ± 7.17	

Subgroup analysis was performed for the outcomes according to different factors (age, type of trochlear dysplasia, and TT‐TG distance); outcomes (Tegner, Kujala, and IKDC scores) in the table were reported as mean with SD. IKDC, International Knee Documentation Committee; TT‐TG, tibia tuberosity‐trochlear groove.

By analyzing the functional scores at the last follow‐up, it was evident that patients younger than 20 years old (13/20) had the worst Kujala (83.46 ± 14.56 *vs* 90.84 ± 7.74, *P* = 0.02) and IKDC scores (83.49 ± 17.35 *vs* 92.46 ± 9.28, *P* = 0.04). Fourteen patients were classified as having type A trochlear dysplasia, and 6 patients type B, but no significant difference in functional scores was found in the two types. Ten patients had higher TT‐TG distance (>20 mm), but no significant difference was found between patients with normal and higher TT‐TG distance.

## Discussion

The main findings of the present study were the improved clinical outcomes for patients with recurrent patellar dislocation after the combined operations at an average of 18 months’ follow‐up. The subjective function scores, VAS scores, Kujala scores, and IKDC scores in the 20 patients were significantly improved. The reduction of the FAA demonstrated that internal femoral rotation was corrected by the surgery. In addition, the radiographic assessments, TT‐TG distance, patellar tilt, and congruence angle, which represented the patellar congruence, were significantly improved after the surgery. No redislocation and severe sustained knee pain had was occurring at follow‐up.

The etiology of patellar instability was assumed to be multifactorial. Anatomical predispositions for the patellar dislocation have been well studied, such as increased tubercle tuberosity‐trochlear groove (TT‐TG) distance, patellar alta, trochlear dysplasia, and genu valgus. The osseous deformities have been treated as the primary targets of the corrective operations and the value of rotational alignments in the lower limbs have been underestimated until now. Researchers have hypothesized that excessive femoral internal rotation could be the primary risk factor for patellar dislocation^5^, ^12^. However, few studies have demonstrated the effectiveness of the combined surgery including femoral derotation osteotomy. In this study, the rotational malalignment in the femur was considered the leading cause of patellar dislocation. Therefore, derotation osteotomy was determined as the most effective surgery to address the etiology for RPD patients with excessive femoral anteversion.

Measurements of the femoral rotation were femoral anteversion angle. In previous studies, the normal range of FAA in the population was stated as around 10°–15° or 15°–20°, which was determined using different techniques^22^, ^23^. The increased internal rotation of the distal femur theoretically increased the relative distance of the trochlear groove and tibial tubercle, leading to a higher TT‐TG distance. Moreover, the Q‐angle was also increased by the internal rotation, which increased the lateral force vector and lateralized the patellar tracking compared to the healthy controls. The TT‐TG distance, over 20 mm pathologically, was the proper indication for the tibial tubercle osteotomy (TTO). However, in this study, we did not conduct TTO to correct the tibial tubercle position for patients. The mean TT‐TG distance of these patients was 22.17 mm and decreased to 20.42 mm after the surgery, which was close to that of healthy controls. The external rotation of the trochlear groove through the derotation osteotomy might account for the decrease in the TT‐TG distance.

In the past fifteen years, soft tissue surgery for restoring the medial patellofemoral ligaments and medial retinaculum has achieved excellent outcomes for patellar instability. In previous studies, better clinical outcomes were obtained using the combined surgery to restore the soft tissue and osseous structure. In the patients in this study, the medial soft tissue was slack and could not maintain the normal patellar tracking in the knee flexion, and the patellar tilt and congruence angle were far beyond the average values in the radiological images, which implied insufficient force in restricting patellar tracking. The medial retinaculum plasty was performed as described in our previous studies in which excellent clinical outcomes had been reported by mid‐term follow‐up^24^, ^25^. In this study, the advantage of the medial retinaculum plasty was direct repair of the medial soft tissue on the femoral side instead of preparing the tendon graft. In addition, the medial retinaculum plasty simplified the surgery by avoiding potential interference between the femoral tunnel for ligament reconstruction and screws of the femoral plate. In this study, the patellar congruence was significantly improved in the radiological assessments at follow‐up. The patellar tilt and congruence angle in this study improved by 14.63° and 16.66° on average. Moreover, there was no dislocation at follow‐up.

According to the findings of Imhoff FB *et al* 26, Frings *et al* 27, and Nelitz *et al* 28, comparable improvements in knee function were observed in this study for combined distal femoral osteotomy and medial retinaculum plasty. At the last follow‐up, the average Kujala score and IKDC score were significantly improved compared to before the surgery, obtaining almost the same levels compared with previous studies using other combined operations. The improved clinical scores of these patients also indicated that the healing of the osteotomy line did not cause additional risks for the recovery of knee motion. Although the procedure of osteotomy might account for 4 of the 20 patients’ delayed rehabilitation due to knee pain and flexion limitation, all patients were satisfied with the recovery of knee function.

Furthermore, we analyzed the effects of different variables on the postoperative outcomes and significant difference was only found in age. A possible reason why younger patients (≤20 years) had worse function recovery may be the weaker muscle strength than for older patients. Moreover, we did not include the cases of severe trochlear dysplasia (Dejour C and D), which was the indication of trochleoplasty. The TT‐TG distance was proved to not be an influencing factor in this study due to the inclusion criteria. The limitations of the study should be addressed. First, the design of this study was a retrospective case series. Further studies with additional groups should be undertaken to evaluate the effects of combined surgery including femoral derotation osteotomy. Second, longer follow‐up should be carried out to observe the long‐term clinical outcomes of the surgery.

### *Conclusion*

In conclusion, the key finding of this study was the favorable outcomes in correcting rotational malalignments in RPD patients. Good knee function, pain relief, and improved patellofemoral congruence were achieved with combined femoral derotation osteotomy and medial retinaculum plasty. The combined operations serve as an ideal treatment strategy not only for recurrent patellar dislocation but also for addressing the primary risk factors.
